# Transformation of PVP coated silver nanoparticles in a simulated wastewater treatment process and the effect on microbial communities

**DOI:** 10.1186/1752-153X-7-46

**Published:** 2013-03-04

**Authors:** Casey L Doolette, Mike J McLaughlin, Jason K Kirby, Damien J Batstone, Hugh H Harris, Huoqing Ge, Geert Cornelis

**Affiliations:** 1 School of Agriculture Food & Wine, The University of Adelaide, PMB 1, Glen Osmond, SA, 5064, Australia; 2 CSIRO Land and Water, Environmental Biogeochemistry Program, Advanced Materials Transformational Capability Platform-Nanosafety, Waite Campus, Waite Road, Urrbrae, SA, 5064, Australia; 3 Advanced Water Management Centre, The University of Queensland, St Lucia, Queensland, 4072, Australia; 4 School of Chemistry and Physics, The University of Adelaide, Adelaide, SA, 5005, Australia; 5 Department of Chemistry, The University of Gothenburg, Kemivägen 10, Göteborg, 41296, Sweden

**Keywords:** Silver nanoparticles, Silver sulfide, Wastewater treatment, STEM HAADF, Sequencing batch reactor, Nitrification, Microbial communities, Pyrotag sequencing, Silver speciation, XAS, Synchrotron, Biosolids

## Abstract

**Background:**

Manufactured silver nanoparticles (AgNPs) are one of the most commonly used nanomaterials in consumer goods and consequently their concentrations in wastewater and hence wastewater treatment plants are predicted to increase. We investigated the fate of AgNPs in sludge that was subjected to aerobic and anaerobic treatment and the impact of AgNPs on microbial processes and communities. The initial identification of AgNPs in sludge was carried out using transmission electron microscopy (TEM) with energy dispersive X-ray (EDX) analysis. The solid phase speciation of silver in sludge and wastewater influent was then examined using X-ray absorption spectroscopy (XAS). The effects of transformed AgNPs (mainly Ag-S phases) on nitrification, wastewater microbial populations and, for the first time, methanogenesis was investigated.

**Results:**

Sequencing batch reactor experiments and anaerobic batch tests, both demonstrated that nitrification rate and methane production were not affected by the addition of AgNPs [at 2.5 mg Ag L^-1^ (4.9 g L^-1^ total suspended solids, TSS) and 183.6 mg Ag kg ^-1^ (2.9 g kg^-1^ total solids, TS), respectively].

The low toxicity is most likely due to AgNP sulfidation. XAS analysis showed that sulfur bonded Ag was the dominant Ag species in both aerobic (activated sludge) and anaerobic sludge. In AgNP and AgNO_3_ spiked aerobic sludge, metallic Ag was detected (~15%). However, after anaerobic digestion, Ag(0) was not detected by XAS analysis. Dominant wastewater microbial populations were not affected by AgNPs as determined by DNA extraction and pyrotag sequencing. However, there was a shift in niche populations in both aerobic and anaerobic sludge, with a shift in AgNP treated sludge compared with controls. This is the first time that the impact of transformed AgNPs (mainly Ag-S phases) on anaerobic digestion has been reported.

**Conclusions:**

Silver NPs were transformed to Ag-S phases during activated sludge treatment (prior to anaerobic digestion). Transformed AgNPs, at predicted future Ag wastewater concentrations, did not affect nitrification or methanogenesis. Consequently, AgNPs are very unlikely to affect the efficient functioning of wastewater treatment plants. However, AgNPs may negatively affect sub-dominant wastewater microbial communities.

## Background

Rapid expansion of the nanotechnology industry has occurred over the previous decade. Manufactured nanomaterials (MNMs) encompass a variety of engineered materials, which can be divided into two groups for the sake of clarity: nano-sized particles (having at least two dimensions < 100 nm) and secondly, materials that are not particulate but have nano-sized properties [1] (i.e. enhanced electronic, optical and chemical properties compared to the bulk material). Silver (Ag^0^) nanoparticles (NPs) are the most widely used NPs in both consumer products and in medical applications [2]. The anti-bacterial properties that render AgNPs desirable may lead to increased risks to human and environmental health following release into the environment. The primary exposure pathway of AgNPs into the environment is via wastewater streams. Silver NPs may enter wastewater through the washing of Ag nano-containing textiles [3,4] or plastics [5], or as a result of the use of nano-enhanced outdoor paints [6] and washing machines [7].

Several authors have investigated the fate of manufactured AgNPs in wastewater treatment plants (WWTPs) and have reported that the majority (> 85%) of AgNPs will be captured by biosolids (stabilised sludge) [5,8-11]. Accordingly, the predicted effluent concentrations of AgNP are very low (ng L^-1^) [11], whereas AgNP concentrations in sludge are predicted to be much higher (1 – 6 mg Ag kg^-1^) [11]. Both concentrations are likely to increase as the AgNP producing industry expands. Given this scenario, and the strong anti-bacterial effects of AgNPs, the stages of WWT that are likely to be affected by AgNPs are those that are dependent on the efficient functioning of microbes. Such stages are the aerobic activated sludge process and anaerobic digestion, which proceeds the former process in most WWTPs. There are very few studies that have investigated the impact of AgNPs on both processes in a sequential manner. Given that the transformation of AgNPs is likely during WWT [8,12], it is crucial to understand at what stage transformation occurs so accurate risk assesments can be conducted using AgNPs in realistic forms.

During the activated sludge process, organic nitrogen and phosphorus are removed by various microbial communities. Several studies have investigated the impact of AgNPs on nitrification [9,13-15] and the effects on microbial populations that perform these processes [16,17]. However, results from nitrification studies are divergent with no inhibition [9] and varying degrees of inhibition [14,15,18] observed on nitrification following AgNP addition in WWTPs or bioreactors at concentrations between 0.4 and 1 mg Ag L^-1^.

The observed variation is most likely explained by the differences in input variables. A number of parameters differ between studies, all of which are known to influence AgNP fate and toxicity e.g. intrinsic AgNP properties (size, coating), Ag concentration, sludge/wastewater properties (temperature, ionic strength (IS)), total suspended solids (TSS) and dissolved organic carbon, (DOC)), the type of sludge/wastewater used (realistic or artificial) and general experimental set-up (e.g. light intensity and wavelength which may cause photocatalytic reduction of Ag^+^ and AgNP).

The impact of AgNPs on anaerobic digestion has been less studied than that of nitrification. Methanogenic microorganisms are generally less sensitive to toxicants than aerobic communities. Silver NPs have been shown to have no effect on biogas and methane production at concentrations of 40 and 85 mg Ag L^-1^, [19] and [13], respectively.

The bactericidal mechanism of AgNPs (and Ag^+^) to organisms is only partially understood and debate is ongoing as to the exact means of action [20]. However, there is concern that the same properties that render AgNPs useful as an antimicrobial may also impact WWTP performance by affecting sludge microbial populations. A high diversity of bacterial populations in WWTPs is crucial for successful removal of BOD/COD, SS and biological phosphorus and nitrogen.

The effects of AgNPs on sludge microbial communities have been investigated by a limited number of studies. The model nitrifying bacteria *Nitrosomonas europae* has been shown to be adversely affected by AgNPs at concentrations of 0.3 mg Ag L^-1^[16] and 2 mg Ag L^-1^[17]. These are much higher Ag concentrations than would normally be found in the environment at present. The microbial communities found in anaerobic systems generally have a different response to toxicants compared to aerobic communities and are usually more sensitive to surface active and homeostatic inhibitors and less sensitive to metabolic inhibitors [21] . For example, at very high Ag concentrations (40 mg L^-1^), methanogenic communities (*Methanosaeta* and *Methanomicrobiales)* have been shown to be largely unaffected by AgNP exposure [19]. So far, however, there has only been analysis of dominant microbes, through relatively insensitive techniques such as qPCR, without assessing the impact on subdominant populations as allowed by next generation techniques such as t-RFLP.

This study was undertaken to (i) investigate the effects of Ag and polyvinylpyrrolidone coated (PVP) AgNPs on organic nitrogen removal from wastewater (nitrification) (ii) examine the fate of Ag^+^ and AgNPs during various stages of WWT (iii) investigate the effects of transformed Ag^+^ and AgNP on anaerobic digestion efficiency, and (iv) to determine if dominant and niche microbial community structures in aerobic and anaerobic sludge are impacted by exposure to transformed Ag^+^ and AgNPs using pyrosequencing.

## Results and discussion

### Silver nanoparticle partitioning in the sequence batch reactor process

**Figure 1:**
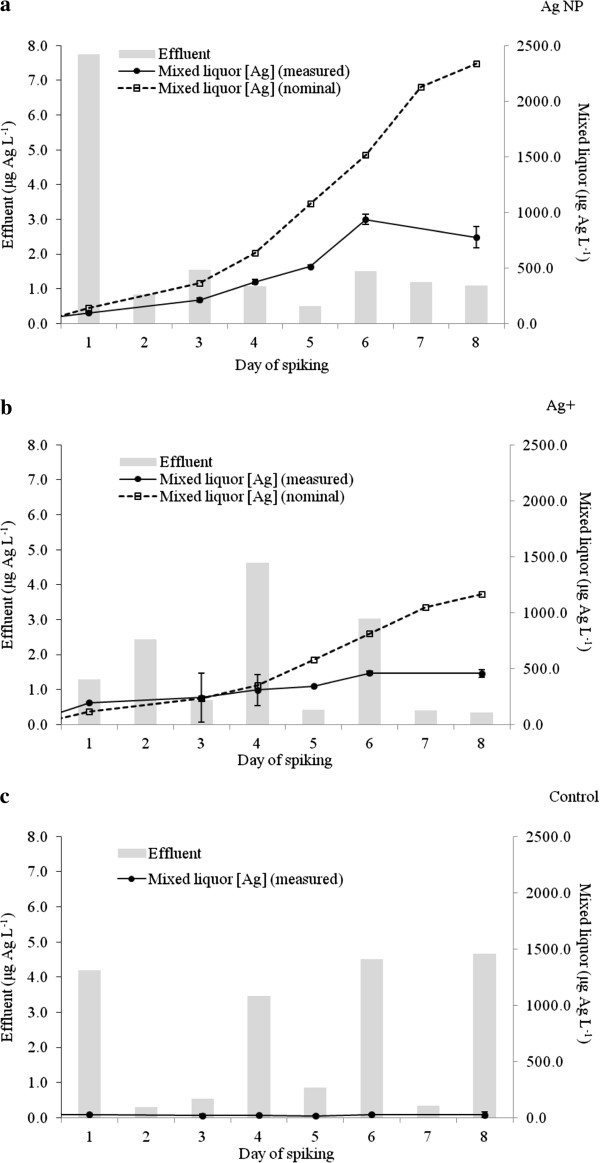
**Silver concentrations in the effluent and the total mass of Ag added to the AgNP dosed (a); Ag**^**+ **^**dosed (b); and, control (c) SBRs.** Less than 1% of added Ag was found in the effluent. Nominal Ag concentrations were calculated from measured Ag spiking solution concentrations. Error bars represent one standard deviation (n = 3).

Measured concentrations of Ag as a function of time in the mixed liquor and effluent are shown in Figure 1. Silver concentrations in the mixed liquor of each sequencing batch reactor (SBR) increased non-linearly during the 10 d aerobic stage. The cumulative concentration of Ag in the mixed liquor was less than the nominal value (taking into account Ag losses with effluent) possibly due to losses of mixed liquor that occurred during sampling for nitrification analysis and during decanting. In addition, Ag losses may have been due to sorption/complexation of Ag/AgNPs onto SBR tubing and container walls.

The effluent concentrations of Ag in the SBR spiked with AgNPs varied from 0.5 μg L^-1^ (day 5) to 7.7 μg L^-1^ (day 1). This corresponds to between 0.1% (for days 5 –8) and 5.4% (day 1) of the total amount of Ag in the mixed liquor (nominal) being removed with the effluent. Similarly, in the Ag^+^ dosed SBR, between 0.1% (days 5 – 8) and 1.1% (day 1) of Ag was released with the effluent. Surprisingly, the Ag concentration range of the effluents collected from the control SBR [0.3 μg L^-1^ (days 2 and 7) to 4.7 μg L^-1^ (day 8)] were within the same range as the effluents collected from the Ag^+^ dosed SBR; 0.3 μg L^-1^ (days 7 and 8) to 4.7 μg L^-1^ (day 4). This can be explained by background Ag concentrations in the influent wastewater (15.0 ± 7.6 μg Ag L^-1^) and activity sludge mixed liquor. Overall, the average (*n* = 8 days, where day 1 and 8 are the first and last days of Ag addition) percentages of Ag in the effluents ± standard deviation (SD) were 0.8 ± 0.1%, 0.4 ± 0.4%, and 2.0 ± 2.8%, for the AgNP, Ag^+^ and control SBRs, respectively. There is large variation in the control as the background Ag concentrations were close to inductively coupled plasma-mass spectrometry (ICP-MS) instrumental detection limits (0.05 μg/L). The results demonstrate that the majority of Ag spiked into SBRs, as AgNPs or ionic Ag^+^ was retained by the sludge.

The partioning results in this study are in agreement with previous studies which have shown that the majority of AgNPs in wastewater will be partitioned to the sludge fraction following wastewater treatment [8-10]. However, the degree to which AgNPs are removed from wastewater has varied between each study. In a pilot WWTP experiment [8], 2.5% of spiked Ag (added as AgNPs stabilised by polyoxyethylene fatty acid ester) was released from the WWTP with the effluent, whereas in a 15 d simulated SBR experiment (0.9 L working volume), citrate coated AgNPs were found to be completely removed from the wastewater [9]. In the literature, the lowest removals of AgNPs from wastewater (88 ± 4%) were recorded from a SBR experiment using synthetic wastewater and AgNPs with an unspecified polymer coating [10].

The observed variations in removal efficiency of AgNPs from the above studies may be due to a number of factors including; the intrinsic properties of the NP (i.e. size, surface charge and capping agent) which in turn are influenced by additional parameters (e.g. mixed liquor pH, chloride concentrations, etc.), method of spiking [16] and perhaps most importantly, the characteristics of the influent wastewater and activated sludge. The TSS content of the influent and activated sludge determines the initial mixed liquor TSS. In the current study, the TSS content (4.5 ± 0.6 g TSS L^-1^) was greater than that used in other studies [8-10](3, 2.4 and 1.8 g TSS L^-1^, respectively). This may explain the high removal efficiency of AgNPs from wastewater (> 99%) which we observed in this study. Most NPs in WWTP sludge is likely to be heteroaggregated with bacteria [10,22-24] but NPs can also be associated with iron oxides or other inorganic particles [23].

At the conclusion of the SBR experiment, sludge Ag concentrations were 418, 168 and 6 mg Ag kg^-1^(TS) for the AgNP, Ag^+^ and control treatments, respectively. The high concentration of Ag in the AgNP sludge is due to the higher Ag concentrations in the AgNP spiking suspensions (mean ± SD; 39 ± 6 mg Ag L^-1^, n = 11) compared to the Ag^+^ solution (mean ± SD; 19.4 ± 0.1 mg Ag L^-1^, n = 3); rather than a greater removal of Ag from the wastewater fraction. Due to logistical limitations, the concentrations of each AgNP suspension could not be determined before spiking as the homogenised NP suspensions degrade after 24 h.

The Ag concentrations of the prepared AgNP suspensions were higher than we had previously achieved and therefore higher than the nominal spiking concentration. The total mass of Ag added to the AgNP and Ag^+^ SBRs, not including background inputs from effluent, was 12.7 mg and 6.4 mg, respectively. The results from the SBR experiments show that the majority of AgNP is partitioned to the solid phase.

### Silver nanoparticle transformation during the sequence batch reactor process as determined using STEM analysis

**Figure 2:**
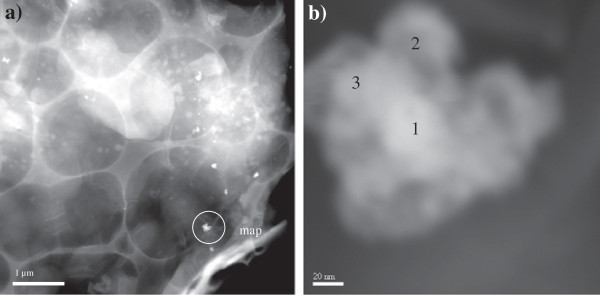
**Characterisation of transformed Ag nanoparticles in aerobic sludge samples using STEM-HAADF.** (**a**) STEM-HAADF image of a typical NP aggregate containing sulfidised nanoscale Ag particles. The bright aggregates are indicative of high *Z* elements (**b**) STEM-HAADF image of a typical Ag aggregate, particles 1–3 were characterised using energy dispersive X-ray (EDX) analysis (see Figure 3). The sludge sample was collected from the AgNP spiked SBR at the end of the experiment SBR experiment.

**Figure 3:**
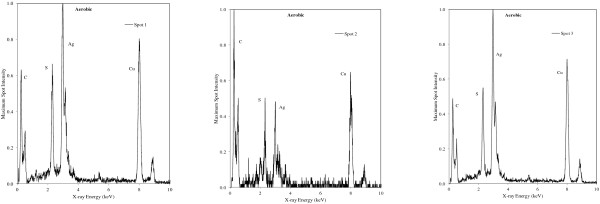
**Energy dispersive X-ray (EDX) spectra of sulfidised silver nanoparticles in aerobic sludge.** Spectra were collected from the specific spots indicated in Figure 2b.

Numerous bright regions were observed in sludge collected from the AgNP dosed SBR (Figure 2a) using scanning transmission electron microscopy (STEM) analysis in high-angle annular dark field (HAADF) mode. Further analysis of the bright spots by energy dispersive X-ray analysis (EDX) confirmed that these regions contained Ag (Figure 3). The STEM image shows aggregates of Ag approximately 100 – 120 nm in diameter (Figure 2b). The higher magnification image (Figure 2b.) shows that each aggregate appears to consist of smaller agglomerated spherical NPs of approximately 40 – 50 nm diameter. EDX analysis of this agglomerate showed that each region consisted of Ag and S with varying ratios. Two regions in the 100 – 200 nm aggregate had a Ag/S ratio of 2:1 (spot 1 and 2), whereas one region contained Ag/S with a 1:1 ratio (spot 3) (Figure 3). The specific cause of NP aggregation requires further investigation. However, it may be attributable to a number of factors including the ionic strength of the mixed liquor and the presence of organic chlorides and minerals. Ionic strength in domestic wastewater is typically < 0.1 M, whereas in anaerobic digesters IS is < 1 M, this may cause NP homocoagulation (see Additional file 1: Table SI.2 for wastewater elemental analysis). Conversely heterocoagulation of NPs may arise from the interaction of AgNPs with organic chlorides and minerals.

Silver sulfide (as α-Ag_2_S) in the nano- size range has previously been identified in sludge [8,12]; however, to determine the crystal phase of the nano-sized particles in this study, further crystallographic investigation is required. At temperatures < 173°C, the monoclinic crystalline form of silver sulfide (α-Ag_2_S) dominates (acanthite). For this phase to exist in the current study, Ag(0) in the original AgNP must be first oxidised to Ag^+^[25,26].

A recent study [25] provided evidence for the direct conversion of AgNPs to Ag_2_S via an oxysulfidation mechanism which was dependent on the presence of small amounts of dissolved O_2._ In the SBR experiment, residual O_2_ did remain during the 110 min anoxic phase. Interestingly, EDX analysis showed that S was present in all nanosized Ag particles identified in the sludge despite the very short anoxic phase relative to the sulfidation reaction times of AgNPs (i.e. > 5 h [8]). Given the very short anoxic phase (105 min), the results suggest that sulfidation of AgNPs in mixed liquor may occur more rapidly than previously shown. Alternatively, AgNP sulfidation in the SBR may have been a gradual process that occurred during successive anoxic phases; i.e. during each anoxic phase a fraction of AgNPs may have been sulfidised until all AgNPs were sulfidised. This pathway is unlikely though as ‘fresh’ AgNPs were added each day.

To the best of our knowledge, the results are the first to identify sulfidised Ag nanosized aggregates in aerobically generated sludge. A previously study that identified Ag_2_S in ‘aerobic’ mixed liquor of a pilot WWTP was not truly representative of an aerated sample because the mixed liquor was first subjected to anaerobic treatment [8]. We therefore suggest that Ag_2_S identified in that aerobic sludge would have been produced during the initial anaerobic treatment because once formed, Ag_2_S is very resistant to oxidation and dissolution of Ag (analogous to other metal oxides [27]). Overall, the results show that in WWTPs, the sulfidation of AgNPs may occur during activated sludge treatment prior to anaerobic digestion.

### Silver speciation in wastewater, activated sludge and anaerobic digestate as determined by synchrotron studies

**Figure 4:**
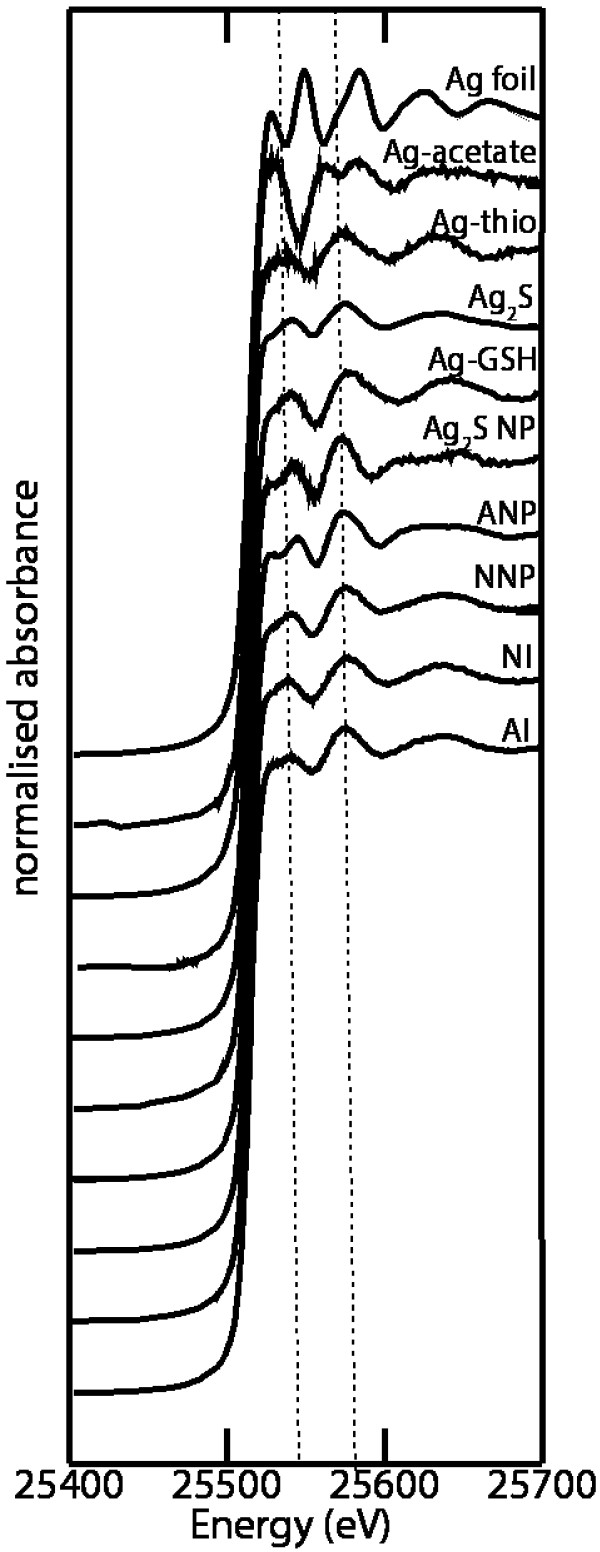
**Silver XANES K-Edge spectra of sludges and the 6 Ag references used for the PCA.** Where ANP = aerobic sludge from the AgNP dosed SBR; NNP = anaerobic sludge from the AgNP treatment; NI = anaerobic sludge dosed with Ag^+^; AI = aerobic sludge from the SBR dosed with Ag^+^; Ag-thio = Ag thiosulfate complex; and, Ag-GSH = Ag glutathione complex. For spectra of the control sludge (collected from the SBR that was not spiked with Ag) see Figure SI.4.

Principal component analysis (PCA) and target transformation identified six standard compounds suitable for the fitting of Ag in sludge samples: Ag_2_S NPs, Ag-acetate, Ag-glutathione (Ag-GSH), Ag-thiosulfate, Ag-foil (Ag^0^) and Ag_2_S (Figure 4). Examination of the XANES spectra of the six target compounds (Figure 4) showed that Ag_2_S NP and Ag-GSH were very similar (also see XANES difference spectra Additional file 1: Figure SI.2b). Therefore, for these Ag standards, their percentage contributions to the sample model fits were combined (Table 1). The two remaining Ag-S models (Ag-GSH and Ag-thiosulfate) are not easily distinguishable from each other by visual inspection of the spectra; however, examination of the difference spectra does show considerable variation (Additional file 1: Figure SI.2b). Furthermore, the identities of Ag-GSH and Ag-glutathione (Ag-GSH) standards were confirmed by EXAFS analysis (data not shown).

**Table 1:** Linear combination fitting analysis of XANES spectra of sludges collected from the SBRs (aerobic), sludges after anaerobic batch tests (anaerobic) and from the short term wastewater experiment

**Sample**	**Ag**_**2**_**S**	**Ag**_**2**_**S NP + Ag-GSH**	**Ag(0)**	**Ag-acetate**	**Ag-thiosulfate**	***Residual***
Aerobic sludges						
Control		40 (5)	20 (3)	19 (3)	23 (4)	0.350
Ag^+^	8 (2)	72 (8)	6 (0.9)	14 (0.9)		0.029
AgNP		85 (4)	15 (4)			0.022
Anaerobic sludges						
Control	39 (9)	24 (7)		27 (4)	11 (6)	0.549
Ag^+^	30 (1)	56 (2)		13 (0.7)		0.015
AgNP	13 (2)	78 (6)		8 (0.8)		0.030
						
Wastewater Experiment						
4 min			100 (0.2)			0.776
24 min			100 (0.2)			0.863
210 min			100 (0.2)			0.927

The proportion of species are presented as percentages with the estimated standard deviation (SD) in parentheses. For the Ag_2_S NP + Ag-GSH column, the SD is the sum of the individual SD’s from each species. The control sludge was collected from the SBR that was not spiked with Ag.

The Ag K-edge XANES spectra of all sludge samples and samples from the wastewater experiment are shown in Figure 4 together with the six references that were used in the linear combination fitting (LCF). The LCF analysis provided good fits to all experimental data (Additional file 1: Figure SI.5). Results show that the dominant Ag species identified in all aerobic and anaerobic sludges was Ag bonded with sulfur (S). The contributions of each standard varied between the different Ag treatments (Ag^+^ vs. AgNP) and also between each treatment process (aerobic vs anaerobic) (Table 1). The exception to this was in the wastewater samples.

The two spectra of AgNP dosed sludge shows that AgNPs were completely transformed during the SBR experiment and again during anaerobic digestion (Figure 4, Table 1). To the best of our knowledge this is the first time that sulfidation of AgNPs has been reported in aerated sludge. The aerobic sample (labelled ANP) was dominated (85%) by sulfidised Ag species with minor amounts of elemental Ag (15%). However, in the anaerobic sample (NNP), elemental Ag was not a significant component. The absence of Ag(0) in the anaerobic AgNP sample is supported by analysis of the corresponding EXAFS spectra (Additional file 1: Figure SI.7) where Ag – Ag bonding was not detected (Table 2).

**Table 2:** Structural parameters of sludges and standards derived from EXAFS analysis

**Sample**	**Shell**	**CN**^**a**^	**R**^**b**^	**σ**^**2c**^
Aerobic sludges				
Ag+	Ag – S	2.0	2.55	0.007
AgNP	Ag – S	1.6	2.51	0.005
	Ag – Ag	2.4	2.89	0.005
	Ag – Ag	3.0	3.09	0.006
	Ag – Ag	2.4	4.98	0.003
Anaerobic sludges				
Ag^+^	Ag – S	2.0	2.48	0.008
AgNP	Ag – S	2.0	2.51	0.005
References				
Ag_2_S NP	Ag – S	1.5	2.52	0.004
	Ag – Ag	3.0	3.06	0.007
Ag-foil	Ag – Ag	12.0	2.86	0.001
Ag-GSH	Ag – S	2.0	2.49	0.003
	Ag – Ag	1.0	3.03	0.007
Ag-thiosulfate	Ag – Ag	1.0	2.52	0.002

^a^ Coordination number, ^b^ Interatomic bond distance (Å), ^c^ Debye-Waller factor.

In the anaerobic AgNP treated sludge, bulk Ag_2_S was detected (13%) whereas in the aerobic sludge it was not detected by XAS. This suggests that the anaerobic digestion process in WWTPs may be vital for the conversion of nano-sized aggregates to bulk forms. The spectral differences that were observed between bulk Ag_2_S and Ag_2_S NPs are a common feature when comparing the XANES spectra of NPs and the bulk. Such effects have been reported for numerous NPs, including Au, CdS, ZnO and Fe_2_O_3_ NPs [28-30]).

Similar sulfidation trends are apparent for the Ag^+^ dosed sludge; the major components of the aerobic and anaerobic sludges were Ag-S coordinated species (80% and 86%, respectively).This was supported by EXAFS analysis which identified Ag– S bonding in each sludge sample (Table 2). The major solid phase speciation changes between aerobic and anaerobic AgNP dosed sludge was the decrease in Ag(0) (15% to 0%) and Ag_2_S NP (40% to 14%) and the subsequent increase in bulk Ag_2_S (8% to 30%). It is most likely that metallic Ag was produced in the Ag^+^ dosed aerobic sludge by photocatalytic reduction of AgNO_3_, or alternatively by reducing agents in the mixed liquor (e.g. hydrogen sulfide, glucose).

Comparison of the XANES spectra for anaerobic Ag^+^ spiked sludge and anaerobic AgNP sludge shows only minor differences. However, much greater differences were found between the aerobic AgNP treated sludge and the anaerobic Ag^+^ sludge (Additional file 1: Figure SI.6).

Silver acetate was identified as a significant component (as determined by the size of the residual following least squares refinement of the model compounds during LCF) in the XANES fitting of the anaerobic AgNP sludge (8%) but not in the aerobic sample. The structure of this standard was not verified by other methods, however, the spectra is significantly different from the Ag-S and Ag(0) standards (Figure 4 & Additional file 1: Figure SI.2b) to be confident that Ag-carboxyl groups are present in the anaerobic sludge sample. In samples where Ag-acetate was detected as a significant component (Table 1), re-fitting the spectra with Ag_2_O produced a poorer quality fit with larger *R*^*2*^ values. Furthermore, when Ag-acetate was excluded from the model, this led to an increase in the fit residuals (see Additional file 1: Table SI. 3 for increased residual values).

Overall the majority of Ag in the AgNP dosed aerobic and anaerobic sludges was sulfidised (85% and 92%, respectively). The results are in agreement with previous studies that have shown sulfidation of AgNPs in sludge [8,12] and highlights the importance of considering Ag speciation in determining the fate and toxicity of AgNPs in terrestrial environments.

The speciation of AgNPs in influent wastewater has not been previously investigated. Based on Ag K-edge XANES results, the results demonstrate that the absence of activated sludge in influent wastewater had a considerable effect on AgNP transformation (Table 1). There was complete transformation of AgNPs to Ag(0) for all wastewater samples; no other species was identified as a significant component in the fit model. Furthermore, there were only subtle differences in the spectra for samples collected initially (4 min after spiking) and after 3.5 h. (Additional file 1: Figure SI.4). The results suggest that when PVP coated AgNPs enter wastewater, their polymer coating will be quickly modified or lost, and aggregation will occur. Additional analysis using the PVP AgNP reference in place of metallic Ag(0) as a target component, produced a poorer fit with a greater residual (3.14 cf. 0.72). It should be noted that the PVP coating of the AgNPs used in the experiments has not been fully characterised (i.e. coating thickness), so this effect may not be observed for all PVP coated NPs. However, it can be concluded that that when AgNPs enter WWTPs, the polymer surface coating may already be modified and AgNPs will no longer be nano in size. The size increase may be caused by a number of factors, including heterocoagulation with natural colloids (e.g. dissolved organic matter and iron and manganese oxyhydroxides) and aggregation due to high ionic strength of the wastewater.

### Effect of silver nanoparticles on nitrification

**Figure 5:**
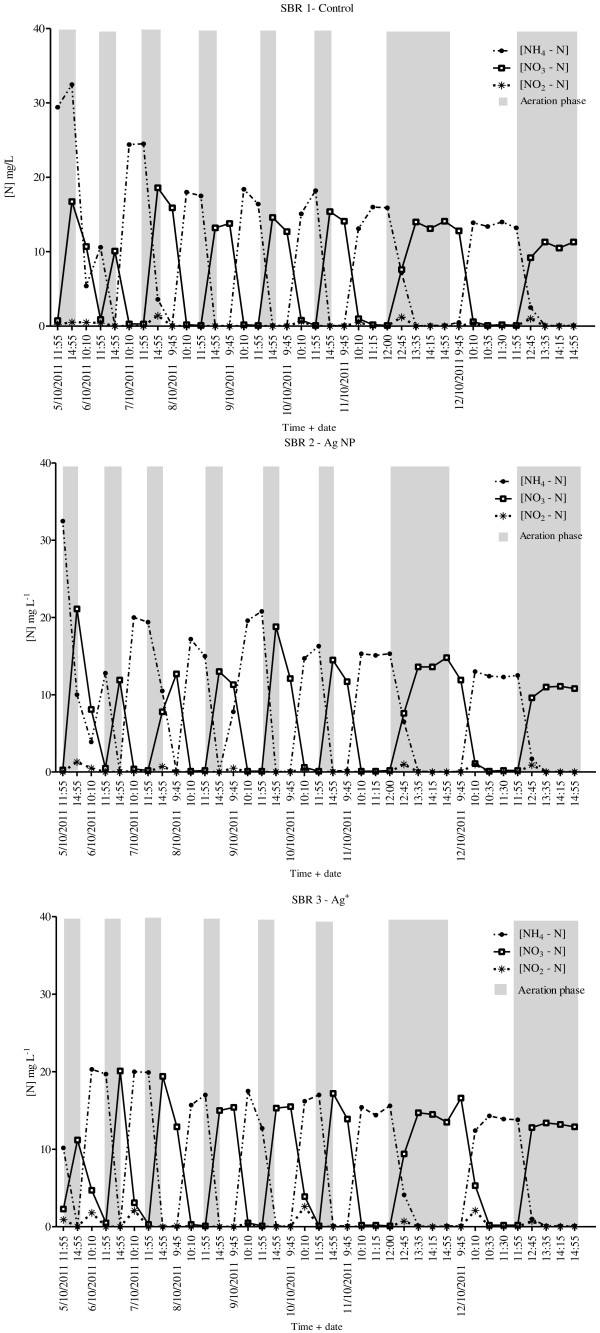
**NH**_**4**_^**+ **^**and NO**_**x **_**profiles of each SBR.** Results are shown from day 2 to day 9 (after spiking). Sample collection commenced on day 1; the second day of SBR operation (results not shown). Samples were collected daily during one cycle (4 cycles in 24 h).

**Figure 6:**
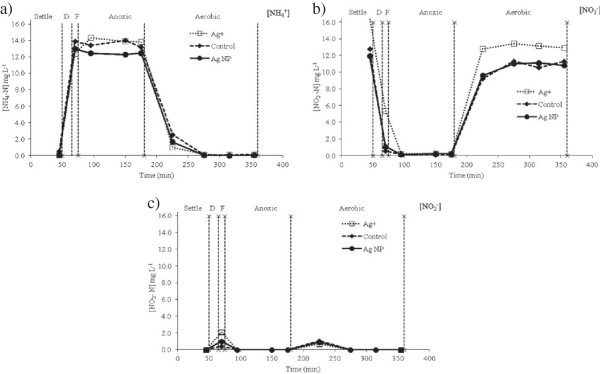
**Variations of (a) NH**_**4**_^**+**^**- N, (b) NO**_**3 **_**– N and (c) NO**_**2 **_**- N profiles during one complete 6 h cycle on day 9.** Where D and F are the decant and feed phases, respectively.

The ammonium (NH_4_^+^) and nitrate/nitrite (NO_x_) profiles of each SBR are illustrated in Figures 5 &6. There was near complete removal of NH_4_^+^ (> 99%) observed from each SBR during each cycle. It should be noted that for the cycle analysed on day 2 for the AgNP dosed SBR only 70% of NH_4_^+^ was removed. In a similar SBR experiment, slight inhibition of nitrifying organisms by AgNPs (citrate capped AgNPs, 0.1 mg Ag L^-1^ of mixed liquor) was also observed on the first day of Ag addition [9]. However, the current results are most likely due to unexpected incomplete mixing of mixed liquor. In the cycle immediately following, complete mixing was resumed, and thus complete NH_4_^+^ removal would be expected as occurred in the other SBRs for day 2. At the beginning of the aeration phase the highest concentrations of NH_4_^+^ were observed, with low variation between each SBR. The maximum concentrations were recorded on different days for the control (day 4; 24.5 mg NH_4_^+^ L^-1^), AgNP (day 6; 20.8 mg NH_4_^+^ L^-1^) and Ag^+^ (day 3; 20.3 mg NH_4_^+^ L^-1^) dosed SBRs. Small amounts of nitrite (NO_2_^-^) were produced in the reactors, however, even during the cycles that had the highest concentrations, NO_2_^-^ was completely converted to nitrate (NO_3_^-^) before the end of the phase.

Nitrification rates were calculated using linear regression over time for two complete cycles on days 8 and 9 and normalised for TSS content (Table 3). The rates were calculated from the initial reduction of NH_4_^+^ at the beginning of the aeration phase (Figure 5). Nitrification occurred rapidly in the first 50 – 60 min of the aerobic phase, and as a result the linear regression is based on 3 time measurements. To support these results, an on-line NHx autoanalyser (YSI, USA) was also used on days 8 and 9 to measure NH_4_^+^ concentrations in the AgNP and Ag^+^ dosed SBRs, respectively (Additional file 1: Figure SI.1). A comparison of the nitrification rates calculated from both analysis methods shows comparable results (Table 3); confirming the accuracy of the chemical data. Probe determined NH_4_^+^ concentrations are between 4.5 mg L^-1^ and 5.4 mg L^-1^ lower than those obtained from chemical analysis (Table 3). This may be due to the close proximity of the probe to the aeration stone in the SBR. Alternatively, the lower pH of the mixed liquor compared to the calibration solutions may have caused a shift of the NH_4_^+^ equilibrium (*NH*_3_ + *H*^+^ ⇌ *NH*_4_^+^) to the left, decreasing the concentration of NH_4_^+^. There was very limited variation in the nitrification rates of all SBRs.

**Table 3:** Nitrification rate for each SBR on days 8 and 9 of the experiment

	**Day 8**	**Day 9**
**SBR**	**mg L**^**-1**^**.h**	**mg L**^**-1**^**.h**
Control	2.4	1.8
Ag^+^	1.5	1.3 (1.2)
AgNP	2.6 (2.2)	1.6

Rates calculated from the on-line NH4 probe are shown in parentheses. All rates have been normalised for TSS content.

Several studies have investigated the effect of AgNPs on nitrification in WWTPs, but results are conflicting [9,14,15,18]. As the sludge matrix is likely to have a major influence on the fate of AgNPs in WWTP, the results from the current experiment are most comparable to those experiments that have used WWTP sludge and activated sludge [9,15]; not synthetic wastewater. In a 15 d simulated SBR experiment [9], NH_4_^+^ removal efficiency was not affected by AgNPs in wastewater (0.5 mg Ag L^-1^), whereas in a short term (12 h) batch test using a synthetic feed solution [15], a 7% decrease in nitrification rate at 1 mg AgNP L^-1^ was recorded. This inhibition may be due to the relatively high DO concentrations (~ 7.2 – mg L^-1^) compared to the more realistic concentrations used in our experiment (1.5 – 2.5 mg L^-1^).

In the current experiment, the complex sludge matrix may have decreased AgNP toxicity for a number of reasons, with two primary factors being the presence of organic matter and the high ionic strength. Organic matter complexes Ag^+^[31,32], which has been linked to AgNP toxicity, whereas high salt concentrations cause NP aggregation which is known to decrease nanoparticle toxicity [33]. In addition, Ag^+^ anion binding may produce very stable products such as AgCl (K_sp,__H2O, 25°C_ = 1.77 x 10^-10^) and Ag_2_S (K_sp,__H2O, 25°C_ = 5.92 x 10^-51^) which will also decrease Ag^+^ bioavailability and hence toxicity.

### Effect of silver nanoparticles on methane production

**Figure 7:**
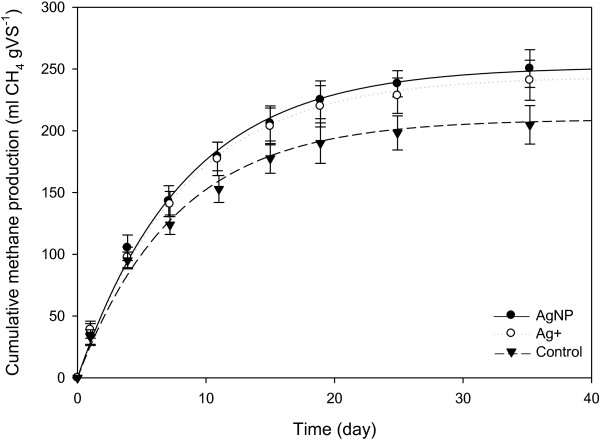
**Cumulative methane produced during the 38 d anaerobic digestion.** All values are blank corrected and the error bars show 95% confidence intervals calculated from triplicate measurements.

The cumulative production of biogas (methane) during anaerobic digestion of the AgNP and Ag^+^ dosed sludges is shown in Figure 7. Based on the calculated anaerobic biodegradability parameters, AgNPs did not have an impact on sludge digestion (Table 4). There was no difference between the methane production of AgNP, Ag^+^ and control sludges at Ag concentrations of 184, 77 and 6.3 mg Ag kg ^-1^. The results concur with previous studies that found methanogenesis was not affected by AgNPs at concentrations < 18.9 mg Ag L^-1^[18] and 40 mg Ag L^-1^[19]. Similarly, for bulk Ag, the rate and extent of methanogenesis in mixed cultures was not affected by either AgNO_3_ or Ag_2_S at concentrations of 100 mg Ag L^-1^[34]. To the best of our knowledge, the results are the first to demonstrate that transformed AgNPs in sludges (present mainly as Ag bonded to S groups) (Table 1), as opposed to ‘pure’ AgNPs, have no effect (at 184 mg Ag kg^-1^) on methanogenic processes which are essential for sludge degradability in WWTPs.

**Table 4:** **Anaerobic biodegradability of each sludge as indicated by degradation extent (*f***_***d,***_**), apparent first order hydrolysis rate coefficient (k**_**hyd**_**) and the estimated methane potential (B**_**0**_**)**

**Treatment**	**k**_**hyd **_**(d**^**-1**^**)**	***f***_**d**_	***B***_***0 ***_***(mL/gVS)***
Control	0.13 ± 0.020	0.31 ± 0.016	195 ± 1
Ag+	0.12 ± 0.014	0.36 ± 0.014	228 ± 9
AgNP	0.12 ± 0.014	0.36 ± 0.014	238 ± 9

### Effect of silver nanoparticles on niche microbial communities

**Figure 8:**
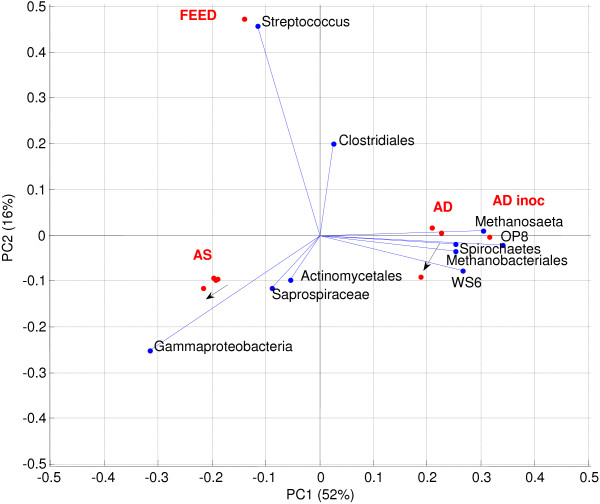
**Overall bi-plot of PCA data showing top 10 OTUs.** All OTUs were used for analysis. The key clusters of Activated sludge - AS (inoculum Ag^+^, and control overlap), Feed, and Anaerobic Digestion (AD) are shown. Arrows show shift from control and Ag^+^ to AgNP communities. Note that AD Inoculum is right-shifted on PC1 compared to the control and Ag^+^ samples.

Following mixed liquor digestion in the three SBRs and anaerobic assays, the diversity of bacterial populations was determined and compared to that of influent wastewater, activity sludge mixed liquor (aerobic inoculum) and anaerobic inoculum. The results from a PCA of the individual data sets shows that all samples could be grouped based on their source (Figure 8). In each case, there was a slight shift from control/Ag^+^ to AgNP.

One dimension could account for 70% of overall variation. Aerobic samples were heavily dominated by a major dominant γ-Proteobacteria 19%, 22% and 21% for the control, AgNP and Ag^+^ aerobic samples, respectively (Figure 8) This is surprising as nitrifiers and phosphate accumulating organisms (PAOs) in activated sludge are usually *β-Proteobacteria* with only a small percentage from the gamma subclass. Removal of organic N is a two-step process where ammonia is initially oxidised to nitrite by ammonia oxidising bacteria (AOB) and then further oxidised to nitrate by nitrite oxidising bacteria (NOB). All AOB belong to two genera each in the *β-Proteobacteria* and γ-*Proteobacteria* phylum, whereas NOB belong to five genera in various classes of the *Proteobacteria*. The primary habitats of *γ-Proteobacteria* are marine environments whereas *β-Proteobacteria* dominate in freshwater systems. The inoculum plant was in a coastal environment (more saline), which could account for this increased dominance.

Ammonia oxidising bacteria are generally more sensitive to toxicants than NOB [16,35]. There was a very minor response to the AgNP treatment, driven mainly by slight shifts in niche populations. Subdominant microbial structure in the Ag^+^ treated sludges was not significantly different to that of the control. What was more surprising was that there was almost no shift in population between the inoculum and Ag^+^ and control. This is surprising as the feed is different, the mode of operation is different (continuous in parent vs sequenced in SBR), and at least 1 nominal sludge age occurred through the study. The pyrosequencing data confirms the nitrification results, in that AgNPs at a concentration of 2.5 mg Ag L^-1^ (2.9 g TS kg^-1^), do not influence the broad microbial population.

**Figure 9:**
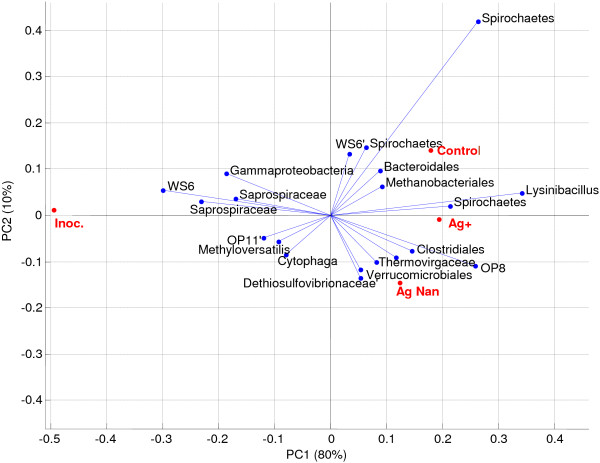
PCA analysis of anaerobic pyrotag results only.

Anaerobic samples indicated that control and Ag^+^ were very similar, but with a large shift from inoculum to batch, and a small shift from control/Ag^+^ to AgNP (Figure 9). This was confirmed through additional PCA analysis on the anaerobic samples only (top 500 OTUs, Hellinger adjusted). This indicated a large shift from inoculum to end BMP, with a dominant WS6 OTU being largely replaced by OP8 (both candidate divisions), and a number of other major OTUs. Silver NPs seemed to cause a subtle shift from Spirochaetes to other organisms. *Archaea* seemed not at all influenced by batch operation, or Ag^+^/AgNP treatment. Our data therefore support those in previous studies [19] indicating no impact of AgNPs on *Archaea* compared to controls (at 20 mg AgNP L^-1^).

What is surprising is the dominance in all anaerobic samples by uncultured division OTUs (Figure 9). The inoculum contained phyla from the uncultured candidate division WS6 (18%), whereas microbial population in anaerobic samples collected after digestion appeared to be dominated (10 – 17%) by organisms from another candidate division (OP8). The cause of this shift in diversity is unclear. The WS6 phyla was first identified in a contaminated aquifer and has since been identified in other environments (e.g. anoxic pond sediment [36], sulfur-rich spring sediments [37], eutrophic estuaries [38], hydrothermal vents [39]) but not in sludges or wastewater. Organisms from the OP8 division have been identified in mangrove sediments [40] and an anaerobic sludge digester, where 1% of the operational taxonomic units (OTUs) were represented by organisms from this division [41]. Broadly speaking, environmental bacterial community structure is regulated by local conditions. Hence, in the BMP test, factors including salinity and nutrient conditions [42] may have differed to that in the tank where inoculum was collected causing the population change.

Sensitive methanogenic microbes (*Methanosaetaceae*) accounted for ~ 11% of the variation in all anaerobic samples, including the inoculum, and was not impacted by the presence of AgNPs (Figure 9). This organism is most sensitive to possible surface active agents [21]. It is highly important that the bacterial population changed so strongly between inoculum and the end of the batch, while the archaeal population seemed untouched. This means that the mode of operation has a strong impact on acidogenic microbial populations but not methanogenic ones. It will be important to further evaluate the role of organisms in candidate divisions, as almost nothing is known of these microbes.

Whilst previous studies have investigated the effects of pure AgNPs on wastewater microbial populations, this is the first time that the influence of transformed AgNPs (primarily as Ag-S species) has been investigated. Moreover, to the best of our knowledge, this is also the first time that transformed AgNPs have been shown to affect niche populations. The results cannot conclusively be attributed to a nano- effect due to the higher spike concentration of Ag in the AgNP treated sludge compared to the Ag^+^ sludge. Yet, XAS analysis of the anaerobic sludges did show a greater percentage of nano sized Ag_2_S in the AgNP treated sludge compared to Ag^+^ dosed sludge (78% cf. 53%, respectively) and a lower percentage of bulk Ag_2_S (13% cf. 30%, respectively). This supports our hypothesis that the observed population changes are attributable to a nano-effect, although further research is required to confirm this hypothesis. Nevertheless, the results still demonstrate that even after their transformation to much less toxic Ag-S species, AgNPs have the potential to impact niche microbial communities but are not likely to impact overall WWTP microbial processes (e.g. nitrification and methanogenesis).

## Conclusions

In our experiments, > 99% of PVP-coated AgNPs were removed from wastewater when subjected to activated sludge digestion. During the SBR experiment and subsequent anaerobic digestion stage, nitrogen removal and methane production (respectively) were not affected by transformed AgNPs.

Pyrosequencing analysis of microbial communities showed that AgNPs and Ag^+^ did not affect the dominant populations of nitrifiers and methanogenic organisms in aerobic and anaerobic generated sludges, respectively. However, in both sludges a subtle shift in niche populations was observed. In the case of aerobic sludge samples, the shift was extremely minor, whilst for anaerobically digested samples there was a much larger shift. Additional studies are required to confirm if this change in population is exclusively a nano- effect.

Two conclusions were drawn from the XAS analysis of sludge: (i) AgNPs were sulfidised during SBR operation followed by near complete sulfidation during anaerobic digestion; and (ii) AgNP dosed anaerobic sludge contained a higher fraction on nano sized Ag_2_S species compared to Ag^+^ dosed sludge. The production of stable Ag-S species may have limited the toxicity of AgNPs towards nitrifiers and methanogenic bacteria as Ag^+^ is believed to be the main toxicity mechanism of AgNPs.

Based on our results, PVP-coated AgNPs will not affect nitrification and methanogenesis during WWT, even in the future with the increasing use of AgNPs. Further investigations are required to confirm whether sub-dominant microbial sludge populations are at risk from AgNP exposure, as this may have long term consequences for the successful operation of WWTPs.

## Methods

### Preparation and characterisation of nanoparticle stock solutions

Polyvinylpyrrolidone (PVP) coated (0.1%) Ag NP powders were purchased from Nanostructured & Amorphous Materials, Inc. (Houston, TX) (10 nm nominal particle size diameter). PVP coated NPs were chosen as they are a very common AgNP capping agent. Thus, their use is realistic of the AgNPs that would be released into wastewater streams. Stock suspensions of AgNPs were prepared by adding the NP powder (0.1 g) to ultrapure deionised water (50 mL, 18.2 Ω) and sonicating (1800 W, 3 min) in an ice bath. The AgNP suspension was then centrifuged (2200 g, 15 min) producing a final stock suspension with an average Ag concentration of 35.7 mg Ag L^-1^ (SD = 5.6 mg Ag L^-1^, n = 11), 8% of which was dissolved ionic Ag^+^[43]. The AgNP stock suspensions were prepared daily (30 – 60 min before spiking). Total Ag concentrations of the NP spiking solutions were determined by an open-vessel acid digestion (HNO_3_, 70%) method as described previously [43].

The AgNP suspensions prepared with this method have been extensively characterized previously using dynamic light scattering (DLS, Malvern Zetasizer), transmission electron microscopy (TEM, Phillips CM200 at 120 keV) and X-ray diffraction analysis [44]. The particle size distribution has also been investigated using disk centrifuge analysis (CPS Instruments disc centrifuge 24000 UHR). In summary, the average particle diameter was between 40 nm with < 8% of Ag existing as dissolved Ag^+^ (Additional file 1: Table SI.1 for complete NP characterisation) [44]. Previous work [44] (using the same method and batch of nanoparticles) has showed that the volumetric diameters of the AgNPs observed using TEM corresponded with the crystallite size determined from X-Ray diffraction analysis, the hydrodynamic diameter (d_h_) observed using DLS and the Stokes diameter as found using disk centrifugation.

### Set-up and operation of sequencing batch reactors

Three individual SBRs (control, Ag^+^ and AgNPs) with a working volume of 5 L and an initial TSS of 6.0 g L^-1^ were prepared with 0.9 L of activity sludge mixed liquor (TS = 35.3 g L^-1^) and 4.1 L of influent wastewater. Activated biological nutrient removal (BNR) sludge was collected from an activated sludge wastewater treatment plant (Luggage Point), and influent wastewater was collected from a local domestic wastewater pumping station (Indooroopilly), both located in Brisbane, Queensland, Australia.

Each SBR was operated with four 6 h cycles per day with a hydraulic retention time (HRT) of 15 h. Each cycle consisted of a 3 h aerated aerobic phase, followed by 50 min settling, 15 min decanting, 10 min feeding and 105 min anoxic [low dissolved oxygen (DO)] phases. Feeding, decanting and sampling ports were located at different positions on the reactors. During the 3 h aerobic stage, DO levels were maintained between 1.5 – 2.5 mg L^-1^ by intermittent aeration, controlled with an online DO detector. Following the settling phase, 3 L of supernatant was decanted and 3 L of cold influent wastewater (20°C) was pumped into each SBR. The reactors were continuously stirred with a magnetic stirrer except during settling and decant phases.

Silver (as NPs or AgNO_3_) was added once every 24 h at the beginning of a feed cycle and for the remaining three feed cycles in that 24 hours no Ag was added. Prior to spiking, trace amounts of Ag were recorded in the mixed liquor of each SBR (day 0), (36, 26 and 24 μg Ag L^-1^ for the control, Ag NP and Ag^+^ dosed SBRs, respectively).

Following the 10 day aerobic digestion, sludge was allowed to settle for 2 h and the supernatant decanted. The remaining sludge in each SBR was centrifuged (2 min, 3250 g), to increase the TS concentrations (Table 5), and approximately half was used in the subsequent anaerobic digestion experiment.

**Table 5:** Average characteristics of each sequence batch reactor

**Characteristic**	**Control SBR**	**Ag NP SBR**	**Ag**^**+**^**SBR**
Average pH	6.71 (0.23)	6.67 (0.07)	6.62 (0.09)
Final TSS (g L^-1^)	4.4	4.5	6.3
Final VSS (g L^-1^)	4.0	4.3	5.5
TS after centrifugation of SBR sludge (g kg^-1^)	72.8	60.5	65.4
Sludge [Ag] before spiking (mg Ag L^-1^)	0.04	0.03	0.02
Total Ag added (mg)	0.00	12.73	6.14

Mixed liquor suspended and volatile solids (MLSS and MLVSS, respectively) were analysed every 2 d according to APHA Standard Methods (1992). The chemical characteristics and Ag spiking concentrations of each SBR are given in Table 5.

### Transmission electron microscopy analysis of silver nanoparticles in sludge

Freeze dried sludge was collected at the conclusion of the 10 d SBR process to determine whether physical or chemical transformation of AgNPs had occurred in the AgNP dosed SBR. STEM analysis in HAADF mode was used to investigate the morphology of AgNPs in the sludge, whereas EDX together with TEM was used for elemental analysis. Samples of aerobic sludge were collected at the end of the SBR experiment. Samples were freeze-dried and analysed using a JEOL 2100 TEM operating at 200 kV. Freeze dried samples were ground in methanol using a mortar and pestle and a single drop pipetted onto a 200-mesh copper (Cu) TEM grid with lacey carbon support film and allowed to evaporate at room temperature.

The elemental composition of “bright” NPs/aggregates was investigated using an EDX spectrometer. The TEM was used in scanning mode (STEM) with a high-angle annular dark-field (HAADF) detector. The high angle detector collects transmitted electrons that are scattered (primarily incoherently) to high angles, whilst excluding Bragg (coherent) scattering. The detector provides an image where the contrast is dependent on the approximate square of the atomic number (*Z)*. Accordingly, bright spots in the image correspond to high *Z* elements; making the detection of Ag containing particles in the complex sludge matrix more straight forward than that in a bright-field image.

### Solid phase speciation of silver in sludge using synchrotron radiation

Solid phase speciation of Ag in aerobic and anaerobic sludges was further examined using X-ray absorption spectroscopy (XAS); specifically X-ray absorption near edge structure (XANES) and extended X-ray absorption fine structure (EXAFS) analysis.

Silver K-edge X-ray absorption spectra were recorded on the XAS beamline at the Australian Synchrotron (AS), Melbourne, Australia. The 3 GeV electron beam was maintained at a current of 200 mA in top-up during the sample analysis. The X-ray beam was tuned with a Si (311) monochromator in the energy ranges of 25312–25492 eV for pre-edge (10 eV steps), 25492–25562 eV XANES region (0.5 eV steps) and then 0.035 Å^–1^ steps in k-space for EXAFS. A metallic Ag foil, recorded in transmission mode downstream of the sample, was used as an internal standard to calibrate the energy scale to the first peak of the first derivative of the Ag edge (25515 eV). Spectra of the samples were recorded in fluorescence mode on a 100-pixel Ge detector array at 90^o^ to the incident beam (Canberra/UniSys).

Freeze-dried sludges (aerobic and anaerobic) were finely ground to a homogenous powder and compressed into pellets with a hand press. Samples that had a high Ag concentration were diluted with cellulose material (Sigma-Aldrich). All samples were cooled to ~10 K in a Cryo Industries (Manchester, New Hampshire, USA) cryostat. One to eight scans per sample were collected for XANES spectra and 14 scans per sample were collected for EXAFS spectra. Reference materials measured at the XAS beamline included PVP-coated AgNPs (Nanoamor), AgNO_3_, Ag_2_S, Ag_2_O, AgCl, Ag_2_CO_3,_ and Ag_2_SO_4_ (all purchased from Sigma Aldrich). Additional standards were prepared the day of analysis and stored in the dark until use; Ag_2_PO_4_, Ag-goethite, Ag-kaolinite, Ag-humic acid complex, Ag-fulvic acid complex, Ag thiosulfate (STS), Ag-acetate, Ag-glutathione (Ag-GSH) and Ag_2_S NPs. (See Supporting Information for synthesis and preparation of all Ag standards).

### Solid phase speciation of silver nanoparticles in wastewater using synchrotron radiation

A short term exposure experiment (3 h) was undertaken to examine the potential rapid transformation/reactions of AgNPs in wastewater (in the absence of activated sludge) using synchrotron based XAS. PVP coated AgNPs were spiked into wastewater (500 mL) to a final concentration of 5.4 mg Ag L^-1^. The wastewater was the same as that which was used in the SBR study. The AgNP-wastewater suspension was stirred continuously for 210 min and the DO concentration was measured with an online DO detector (7.4 mg O_2_ L^-1^ to 7.1 mg O_2_ L^-1^). Approximately one mL of the suspension was collected at t = 4, 10, 24, 45, 60, 94 and 210 min after the addition of AgNPs. Each sample was collected using a two mL glass syringe and injected directly into a leucite cuvette, secured with Kapton tape, immediately flash frozen in liquid N_2_ and stored in dry ice until XAS analysis.

### XAS data analysis

The chemical speciation of each sample was determined by fitting a linear combination of model compounds to each XANES spectrum (Additional file 1: Figure SI.3). The number of components in the sample XANES spectra was determined using principal component analysis (PCA) of all sample spectra, followed by target transformation to identify the most likely components of the spectra from a model compound library. The number of components to fit were chosen from the eigenvalues from the PCA and visual inspection of the plot of eigenvectors.

Linear combinations of the six spectra were fitted to each sample spectrum with the best fit to the experimental spectrum achieved by least squares refinement of the model compounds to the experimental spectrum. The best fits were improved by the removal of small components (< 1%) and the adequacy judged by the size of the residual and visual inspection to ensure that all features were accounted for.

Calibration, averaging and background subtraction of all spectra and principle component, target and multiple linear regression analyses of XANES spectra were performed using EXAFSPAK software package (G.N. George, SSRL).

### Investigation of nitrification inhibition and silver partioning during aerobic digestion

For nitrification analysis, homogenous mixed liquor samples were collected daily at the end of feed, anoxic, aerobic and settling phases during one 6 h cycle for the first 7 d of SBR operation. On days 9 and 10, more frequent sampling was conducted during the aerobic and anaerobic phases. Samples were filtered (< 0.22 μm) and stored at 4°C until analysis.

The choice of Ag spiking rate was a compromise between realistic environmental exposure concentrations [11], previous partitioning studies of Ag NP in wastewater [8,18] and instrumental detection limits. Taking these factors into account, the aim was to produce sludges with a final concentration of ~100 mg Ag kg^-1^ TS for the Ag^+^ and AgNP treatments.

The SBRs were operated for 24 h before spiking to allow for equilibration of the mixed liquor. Reactors 2 and 3 received the AgNP and Ag^+^ (as silver nitrate (AgNO_3_)) treatments, respectively. Reactor 1 was assigned the control SBR and received ultrapure deionised water (Millipore) at each spiking event in order to maintain a consistent volume for all three reactors. Treatments were added once daily, for 8 d, to each SBR at the beginning of the aeration phase by pipetting the spiking solution directly into the reactor. Each reactor received a total of 330 mL of the assigned spiking solution (nominal concentration for AgNO_3_ and AgNP suspensions = 20 mg Ag L^-1^). The Ag concentration of the ultrapure deionised water used in the control SBR was below the limit of detection for ICP-MS analysis (< 0.05 μg L^-1^). Reactors were operated for a total of 10 d.

For silver analysis, mixed liquor (10 mL) and effluent (35 mL) samples were collected once daily from each SBR; 3 and 5.5 h after spiking, respectively. Samples were acidified and stored at 4°C before subsequent digestion and analysis for total Ag by ICP-MS.

### Anaerobic digestion and biomethane potential test

The effect of AgNPs on anaerobic digestion was assessed using a biomethane potential (BMP) test as previously described [45]. Anaerobic biomethane potential tests (BMP) were carried out for AgNPs and Ag^+^ using sludge collected from each SBR at the conclusion of that experiment (all assays in triplicate). The inoculum (activity sludge) had a broad trophic microbial composition to ensure the substrate would not be limited. Blank assays (in triplicate) were used to determine the background methane production from the inoculum.

Aerobically digested sludges from the SBRs (substrate) were diluted to 30 g L^-1^ (TSS) with ultrapure deionised water (Millipore). Substrate (40 g wet) and inoculum (60 g wet) (anaerobic digestate from a municipal WWTP, Brisbane, Queensland) were added to glass serum bottles (160 mL working volume), flushed with high purity N_2_ gas for 3 min (1 L min^-1^), sealed with a butyl rubber stopper and aluminium crimp-cap and stored in a temperature controlled incubator (36°C) for 38 d. Blanks (n = 3) contained inoculum (60 g) and ultrapure deionised water (40 mL) (Millipore). Each assay was performed in triplicate. Once daily methane production had ceased (38 d) the batches were terminated, and analysis of the microbial community was conducted. Biogas volume was measured periodically (initially daily) and the quality (CH_4_, CO_2_, H_2_) was analysed using gas chromatography, with a thermal conductivity detector (Perkin Elmer). Confidence intervals (95%) were calculated from triplicate measurements and were ≤ 0.02 g COD d^-1^ for all samples. Excess CH_4_ was vented from each serum bottle periodically via syringe and measured by liquid displacement.

### Microbial community analysis: DNA extraction and 16 s Pyrotag Analysis

To assess the potential impact of AgNPs on microbial diversity of sludge samples (aerobic and anaerobic), a massive parallel sequencing approach using pyrotag sequencing was used. Microbial diversity analysis was conducted on samples of activated sludge mixed liquor (Luggage Point WWTP), feed (Indooroopilly pumping station), sludge after aerobic digestion (control, Ag^+^ and AgNP), anaerobic inoculum (Luggage Point WWTP) and anaerobic digestate (control, Ag^+^, and Ag NP).

Community genomic DNA from the anodic biofilms were extracted using FastDNA SPIN for Soil kit (MP Biomedicals, USA) and Fastprep beadbeating machine (Bio101, USA) according to the manufacturer’s protocol. The 3’ region of the 16S/18S rRNA gene was targeted using universal primers 926 F (5’-AAACTYAAAKGAATTGACGG-3’) and 1392R (5’-ACGGGCGGTGTGTRC-3’). Primer sequences were modified by the addition of Roche 454 adaptor 1 or 2 sequences and unique 5 bp barcodes at the 5’ end of the primer (sequences not shown) [46,47]. DNA concentration and purity was then determined by gel electrophoresis on 1% agarose gel and spectrophotometrically using the NanoDrop ND-1000 (Thermo Fisher Scientific, USA). DNA was lyophilised using Savant SpeedVac Concentrator SVC100H (Thermo Fisher Scientific, USA) and submitted to the Australian Centre for Ecogenomics (ACE) for 16 s rRNA gene pyrotag sequencing on the Genome Sequencer FLX Titanium platform (Roche, USA). Pyrotag sequences were processed using Pyrotagger [48], and QIIME with correction via ACACIA. Operational taxonomic unit (OTU) tables were normalised, and a square root (Hellinger) transformation was applied to emphasise comparison of niche populations over dominants. A principal components analysis was then performed on the square root (Hellinger adjusted) normalised OTU table using Matlab (princomp command), and results visualised using biplot.

### Chemical analysis of silver spiking solutions, mixed liquor, effluent, and sludge

Silver concentrations of the AgNP spiking solutions were determined using an open-vessel acid digestion (HNO_3_, 70%) method as previously described [43].

Effluent and mixed liquor samples were analysed for Ag following microwave digestion in *aqua regia* according to the method used for wastewater previously [8]. Sludge samples (aerobic and anaerobic) were first dried at 40°C to constant weight, and then allowed to react with H_2_O_2_ before using the same *aqua regia* digestion method. Silver concentrations in all digested solutions were determined using ICP-MS (Agilent 7500ce) and He_(g)_ as a collision gas (4 mL min^-1^) and monitoring Ag at *m/z* 107 and 109.

Filtered mixed liquor was analysed for NH_4_^+^, NO_2_^-^ and NO_3_^-^ using a Lachat QuikChem8000 Flow Injection Analyser.

## Abbreviations

AgNPs: Silver nanoparticles; TEM: Transmission electron microscopy; EDX: Energy dispersive X-ray analysis; XAS: X-ray absorption spectroscopy; TSS: Total suspended solids; TS: Total solids; MNM: Manufactured nanomaterials; PVP: Polyvinylpyrrolidone; SBR: Sequencing batch reactor; DOC: Dissolved organic carbon; WWTP: Wastewater treatment plant; DLS: Dynamic light scattering; BNR: Biological nutrient removal; DO: Dissolved oxygen; HRT: Hydraulic retention time; OTU: Operational taxonomic units; SD: Standard deviation; STEM: Scanning transmission electron microscopy; HAADF: High angle annular dark field; PCA: Principal component analysis; LCF: Linear combination fitting; XANES: X-ray absorption near edge spectroscopy; EXAFS: Extended X-ray absorption fine structure; AS: Australian synchrotron; Ag+: Dissolved ionic silver; Ag0: Elemental silver; NH4+: Ammonium; NO3-: Nitrate; NO2-: Nitrite; AgNO3: Silver nitrate; Ag-GSH: Silver glutathione complex; Ag2S NP: Silver sulfide nanoparticles; Ag-thio: Silver thiosulfate; Ag-HA: Silver – Humic acid; Ag-FA: Silver – Fulvic acid

## Competing interests

Authors declare that they have no competing interests.

## Authors’ contributions

CD: designed and conducted the experiments, interpreted results and wrote the manuscript. MJM: designed the experiments, interpreted results and participated in manuscript preparation. JK: designed the experiments, interpreted results and participated in manuscript preparation. DJB: assisted in experimental design, interpreted results and participated in manuscript preparation. HHH: conducted XAS experiments, participated in XAS data analysis, interpreted XAS results and participated in manuscript preparation. HG: Set-up the SBR experiments, conducted the anaerobic batch test and assisted in analysis of results. GC: Participated in experimental design, was involved in useful discussions and participated in manuscript preparation. All authors read and approved the final manuscript.

## Supplementary Material

Additional file 1 **The following additional information data are available with the online version of this paper in Additional file 1.** Methods for synthesis and preparation of synchrotron standards and sample preparation. **Table SI.1**. Characteristics of the silver nanoparticles (AgNPs) and AgNP stock suspensions. **Figure SI.1**. NH_4_ – N profiles of the AgNP and Ag^+^ dosed SBRs as measured by the NH_4_^+^ on-line detector. **Table SI.2**. The concentration of major and trace elements in the influent wastewater **Figure SI.2**. Difference XANES spectra of sludge and various Ag references used in LCF analysis. **Figure SI.3**. Ag K-Edge XANES spectra of all reference materials. **Figure SI.4**. Ag K-Edge XANES spectra of aerobic and anaerobic control sludges and wastewater from the influent experiment. **Figure SI.5**. Bulk silver (Ag) X-ray absorption near-edge spectroscopy (XANES) of sludge collected from the SBRs (a-c) and after the anaerobic batch test (d-f). **Figure SI.6**. Ag K-edge XANES spectra showing the considerable difference between aerobic sludge dosed with AgNP, and anaerobic sludge dosed with Ag^+^ or AgNP. **Figure SI.7**. k^3^-weighted Ag K-edge EXAFS spectra of sludges and their respective phase-corrected Fourier transforms. **Table SI.3**. The higher residual values that resulted from the exclusion of Ag-acetate from the linear combination fitting analysis of XANES spectra of sludges. Click here for file
