# Supplementary material for: Transformation of PVP coated silver nanoparticles in a simulated wastewater treatment process and the effect on microbial communities
Source: Chem Cent J. 2013 Mar 4;7:46. doi: 10.1186/1752-153X-7-46 (PMC3636095; doi:10.1186/1752-153X-7-46)
Supplement: Additional file 1 — The following additional information data are available with the online version of this paper in Additional file 1. Methods for synthesis and preparation of synchrotron standards and sample preparation. Table SI.1. Characteristics of the silver nanoparticles (AgNPs) and AgNP stock suspensions. Figure SI.1. NH4 – N profiles of the AgNP and Ag+ dosed SBRs as measured by the NH4+ on-line detector. Table SI.2. The concentration of major and trace elements in the influent wastewater Figure SI.2. Difference XANES spectra of sludge and various Ag references used in LCF analysis. Figure SI.3. Ag K-Edge XANES spectra of all reference materials. Figure SI.4. Ag K-Edge XANES spectra of aerobic and anaerobic control sludges and wastewater from the influent experiment. Figure SI.5. Bulk silver (Ag) X-ray absorption near-edge spectroscopy (XANES) of sludge collected from the SBRs (a-c) and after the anaerobic batch test (d-f). Figure SI.6. Ag K-edge XANES spectra showing the considerable difference between aerobic sludge dosed with AgNP, and anaerobic sludge dosed with Ag+ or AgNP. Figure SI.7. k3-weighted Ag K-edge EXAFS spectra of sludges and their respective phase-corrected Fourier transforms. Table SI.3. The higher residual values that resulted from the exclusion of Ag-acetate from the linear combination fitting analysis of XANES spectra of sludges. [file 1752-153X-7-46-S1.docx]

Additional Information

**Synthesis of synchrotron standards and sample preparation**

Aqueous Ag^+^ (1 mM) was prepared by dissolving AgNO_3_ in ultrapure deionised water and storing in the dark until use.

Silver phosphate was precipitated from a homogenous solution by the volatilisation of ammonia as described by [[1](#_ENREF_1)]. Briefly, phosphorus oxide (1.3 mmol of H_3_PO_4_) was combined with ultrapure deionised water (50 mL) in an Erlenmeyer flask, followed by the addition of conc. NH_4_OH (0.5 mL) and NH_4_NO_3_ (25 mmol). A precipitant solution was prepared by adding AgNO_3_ (15 mmol) to conc. NH_4_OH (3 mL) in a beaker and adjusting the volume to 75 mL. The precipitant solution was added to the P_2_O_5_ solution in one portion resulting in a clear solution. The combined solution (pH = 9.8) was heated on a low temperature hot plate for 3 h until the pH was < 7.5 at room temperature. The resulting precipitate was filtered, washed three times with distilled water and dried overnight at 70°C.

Silver thiosulfate (0.02 mM) was prepared by adding an excess of (NH_4_)_2_S_2_O_3_ (8 mL, 0.1M) to a stock solution of AgNO_3_ (2 mL, 0.1M), where the ratio of Ag^+^:S_2_O_3_^2-^ was 1:4. The resulting solution was diluted to 1 mM Ag before XAS analysis

Silver acetate was prepared by adding an excess of acetate (as NaCH_3_COOH) (2 mL, 0.1M) to aqueous Ag^+^ (5 mL, 2 mM) and adjusting the final volume to 10 mL with ultrapure deionised water. The nominal Ag concentration was 1 mM.

Silver glutathione (GSH) (1 mM Ag) was prepared by adding 2 mL of a GSH solution (0.1M) to aqueous Ag^+^ (5 mL, 2 mM) and adjusting the volume to 10 mL with ultrapure deionised water. A flocculent white precipitate formed immediately on mixing the solutions and remained suspended in the solution. The spectrum was recorded from the frozen suspension.

To model the possible complexes that Ag may form in the presence of organic compounds or mineral phases in wastewater, Ag was added to fulvic/humic acid and to kaolinite/goethite, respectively. Aqueous Ag^+^ (1 mL, 2 mM) was added to solutions of fulvic acid (200 mg L^-1^) (Suwanee River fulvic acid) or humic acid (200 mg L^-1^ ) (Sigma Aldrich) to give a final Ag concentration of 1 mM. For the mineral phases, aqueous Ag^+^ (0.5 mL, 7.7 mM) was added to goethite or kaolinite (1 g) to give a concentration of 400 mg Ag kg^-1^. Both spiked mineral samples were homogenised by grinding in a mortar and pestle. Prior to XAS analysis, the samples were again ground, but with the addition of cellulose, and pressed into a disc.

All sludge samples and the following references were also ground with cellulose material and pressed into a disc prior to analysis: AgCl, Ag_2_SO_4_, Ag_2_PO_4_, Ag_2_O and Ag_2_CO_3_. We thank Prof. Enzo Lombi for providing a spectrum of Ag_2_S(s) recorded in PVP at beamline 10ID at the APS.

**Characterisation of silver nanoparticles**

The AgNP suspensions were prepared as previously described [[2](#_ENREF_2)]. All characteristics determined in our previous study are listed in Table S1.

Table SI.1. Characteristics of the silver nanoparticles (AgNPs) and AgNP stock suspensions [[2](#_ENREF_2)].

| Property | Value |
| --- | --- |
| Composition (XRFǂ) | Ag (impurities<0.01%) |
| Crystal structure (XRD‡) | Ag metal (mineral impurities <2 mass%) |
| Crystallite size (XRD) | 41 nm |
| Suspended dh# (DLS§) | 44 nm |
| Number-average suspended Stokes diameter (Disc centrifuge analysis) | 33 nm |
| pHIEP¡ | 3.1 |
| pH in unmodified stock suspensions | 4.2 |
| CCCɤ at pH 4 (NaClO_4_) | 22 mM |
| CCC at pH 8 (NaClO_4_) | 45 mM |
| Coating (nominal) | 0.1% PVP |

ǂ X-ray fluorescence analysis

‡ X-ray diffraction

# Apparent *z*-averaged hydrodynamic diameter

§ Dynamic light scattering

¡ pH at which isoelectric point is reached

ɤ Critical coagulation concentration

***
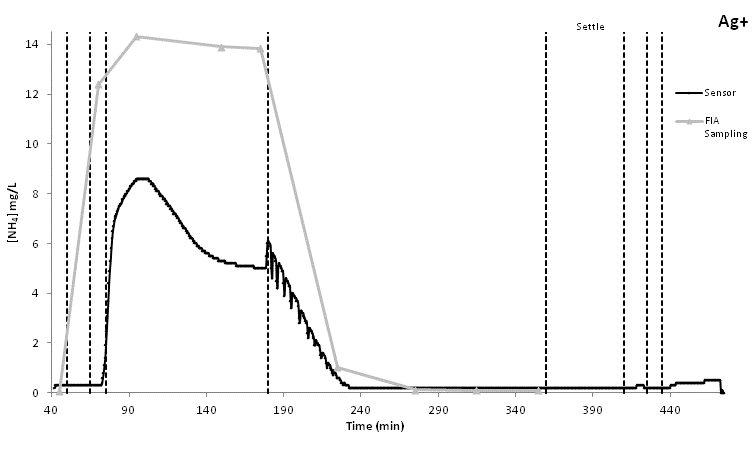

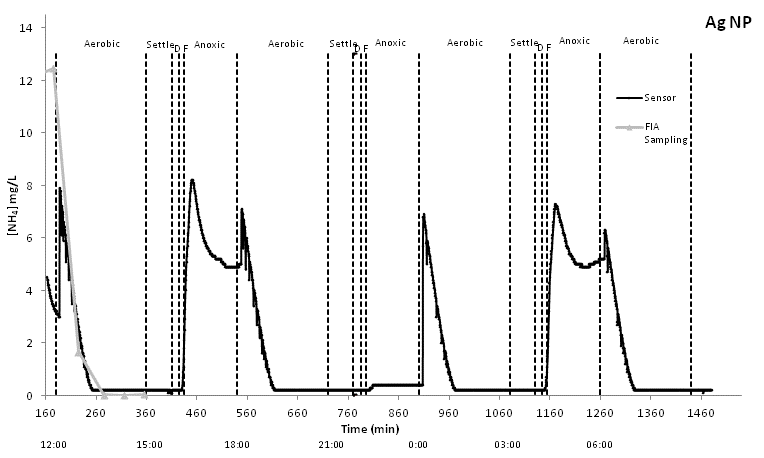
***Fig. SI.1. NH_4_ – N profiles of the AgNP and Ag^+^ dosed SBRs as measured by the NH_4_ on-line detector. Data was recorded for 21 h (~3.5 complete cycles) for the AgNP SBR and 6 h (1 cycle) for the Ag^+^ dosed SBR. The NH_4_ – N concentrations as determined by FIA are also shown.

Table SI.2. The concentration of major and trace elements in the influent wastewater

| Element | Ca | K | Mg | Na | P | S | Sr |
| --- | --- | --- | --- | --- | --- | --- | --- |
| mg/L | 15.3 | 19.6 | 12.6 | 65.8 | 3.2 | 12.5 | 0.1 |

The concentrations of the following elements were below the ICP-OES detection limit (shown in parentheses in mg/L); Al (0.05), As (0.05), B (0.1), Cd (0.05), Co (0.05), Cr(0.05), Cu (0.05), Fe (0.1), Mn (0.05), Mo (0.05), Ni (0.05), Pb (0.05), Sb (0.1), Se (0.05), Si (0.1) and Zn (0.05).


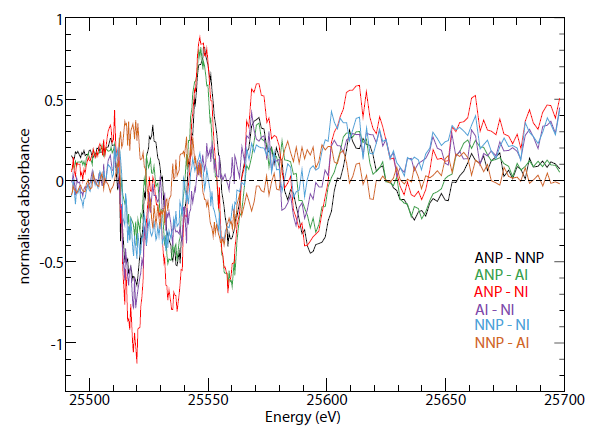

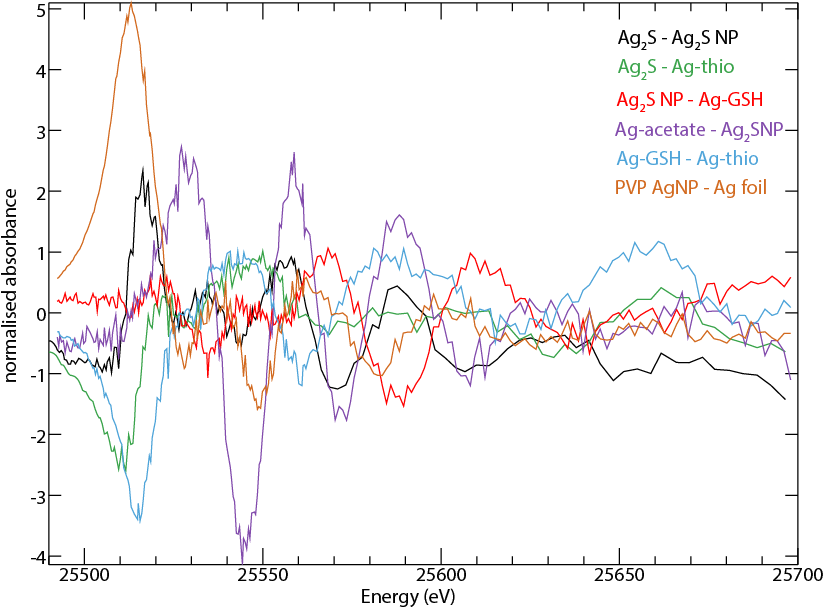
a) b)

Fig. SI.2. Difference XANES spectra of sludges (a) and various Ag references used in LCF analysis. Where ANP = aerobic sludge dosed with AgNPs, NNP = anaerobic sludge dosed with AgNPs, AI = aerobic sludge dosed with Ag^+^, NI = anaerobic sludge dosed with Ag^+^, Ag-thio = Ag-thiosulfate complex and Ag-GSH = Ag-glutathione complex.

Fig. SI.3. Ag K-Edge XANES spectra of all reference materials. Where Ag-FA = Ag adsorbed to fulvic acid; Ag-HA = Ag adsorbed to humic acid; Ag-GSH = Ag-glutathione complex; and, Ag-thio = Ag-thiosulfate complex. The dashed line is to guide the eye.

Fig. SI.4. Ag K-Edge XANES spectra of aerobic and anaerobic control sludges (AC and NC, respectively) and wastewater from the influent experiment. Where WW_4, WW_24 and WW_210 are influent samples collected 4 min, 24 min and 210 min after the addition of AgNPs, respectively.


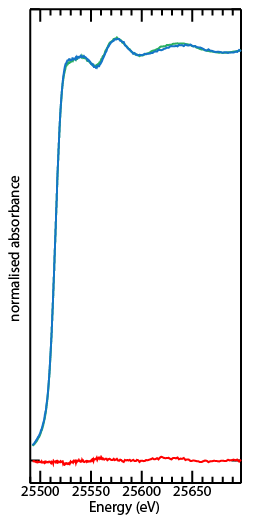

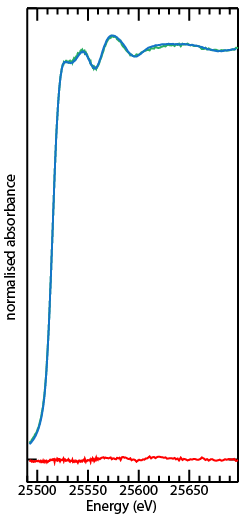
a) b) c)


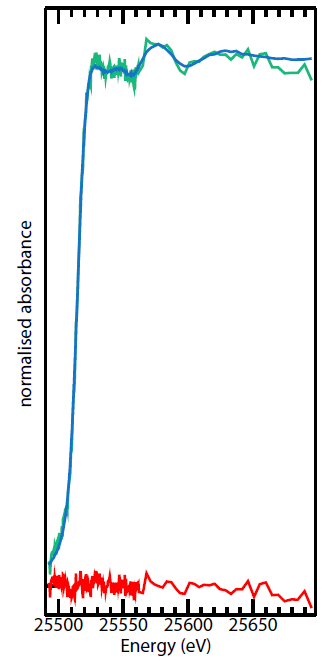


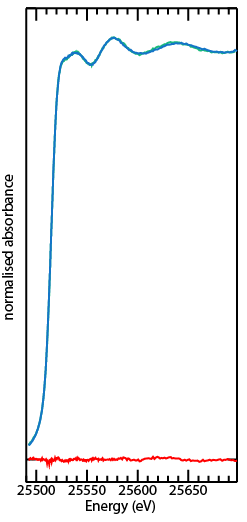

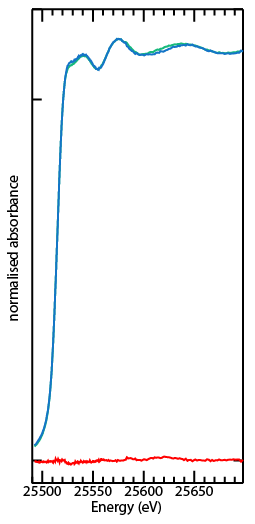
d) e) f)


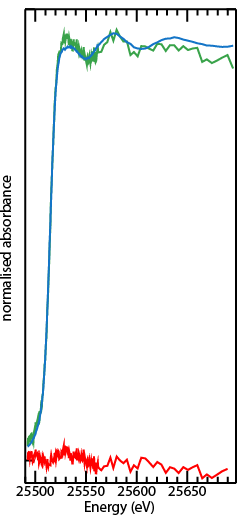


Fig. SI.5. Bulk silver (Ag) X-ray absorption near-edge spectroscopy (XANES) of control, Ag^+^ and AgNP dosed sludges collected from the SBRs (a-c) and after the anaerobic batch test (d-f), respectively. Blue, linear combination fit; green, experimental fit; red, offset residual.


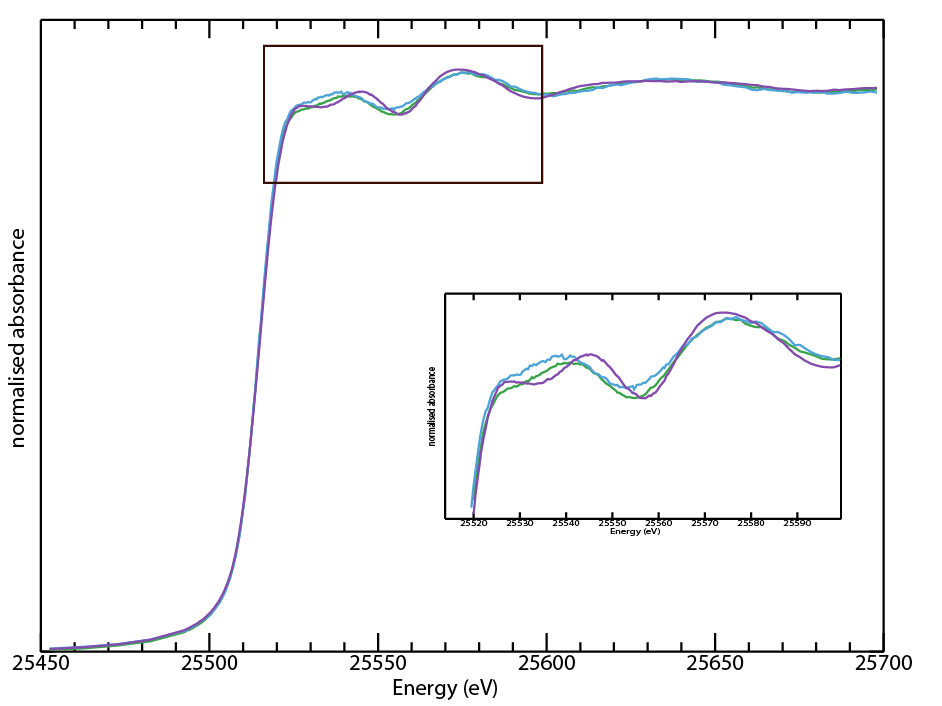


Fig. SI.6. Ag K-edge XANES spectra showing the considerable difference between aerobic sludge dosed with AgNP (purple) and anaerobic sludge dosed with Ag^+^(blue) or AgNP (green).


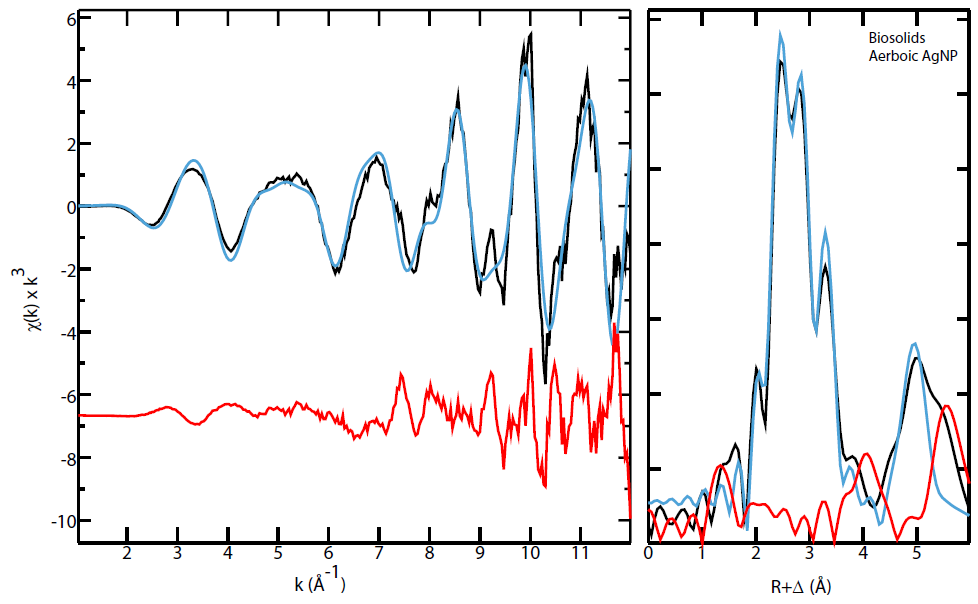

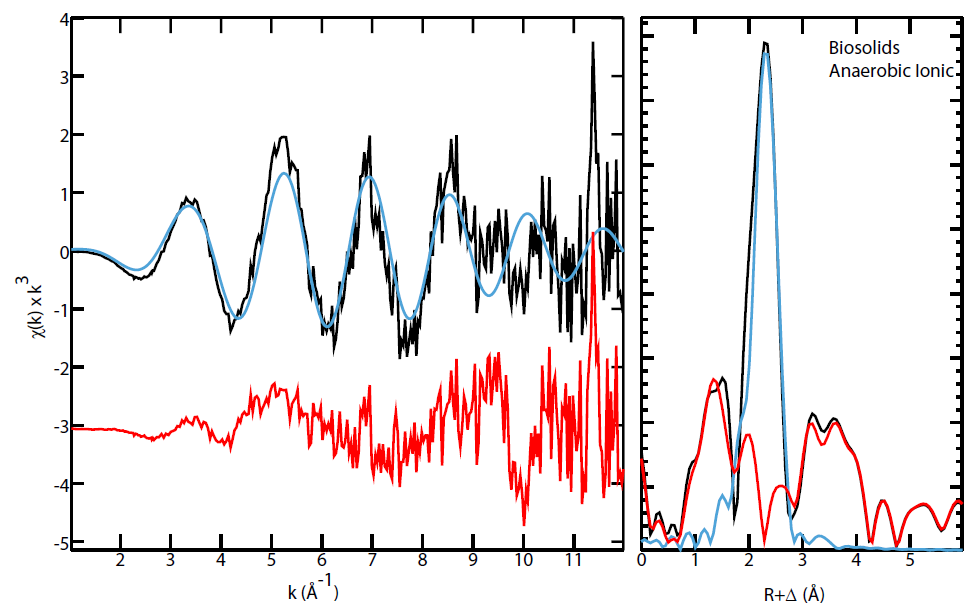


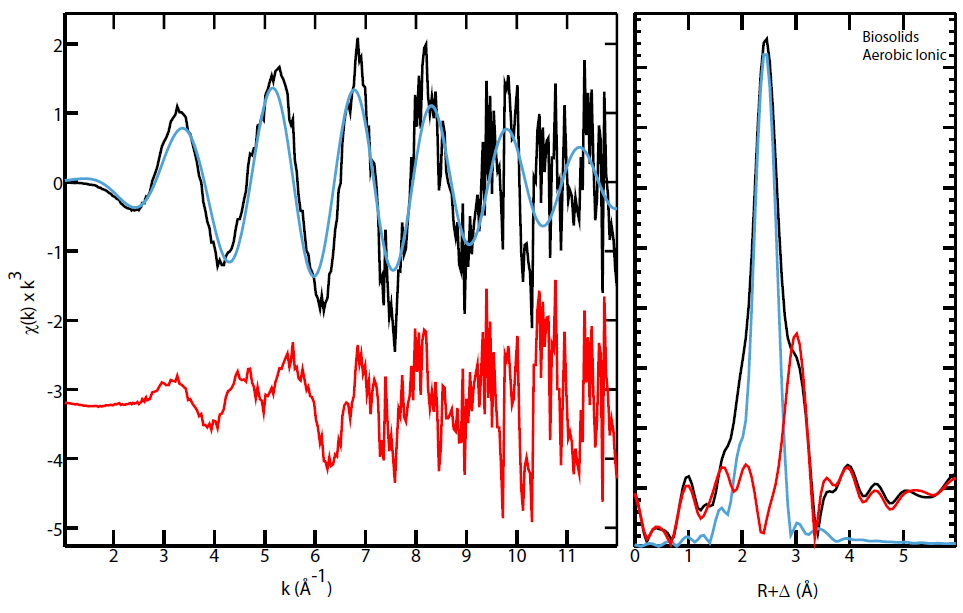


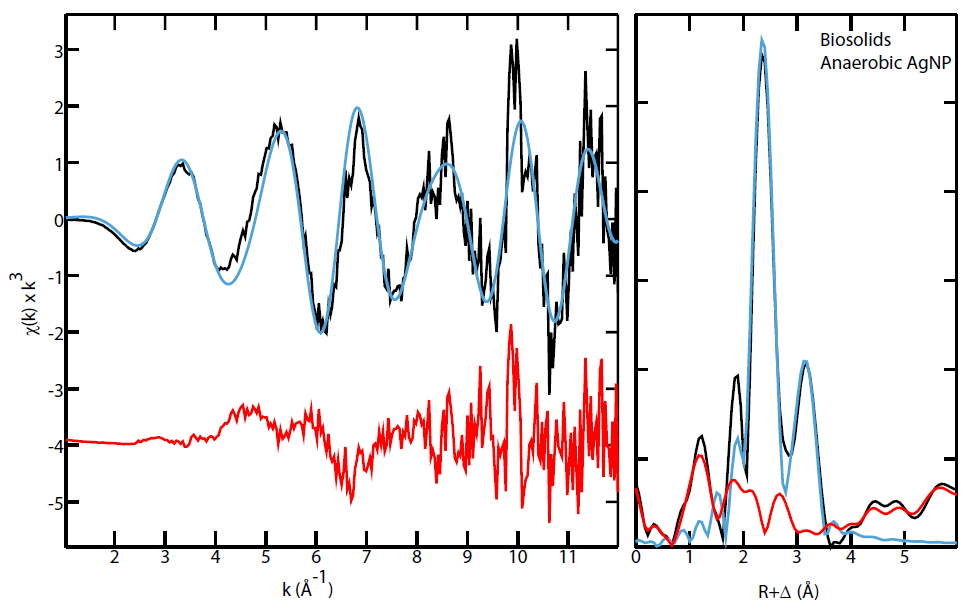


Fig. SI.7. k^3^-weighted Ag K-edge EXAFS spectra of sludges (left panels) and their respective phase-corrected Fourier transforms. Data is shown in black, fit in blue and the residual in red.

Table SI.3. The higher residual values that resulted from the exclusion of Ag-acetate from the linear combination fitting analysis of XANES spectra of sludges. Only those sludge samples that were identified as having Ag-acetate as a significant component in the fit are shown. The proportion of species are presented as percentages with the estimated standard deviation **(SD)** in parentheses**.**

| Sample | *Residual* | *Residual excluding Ag-acetate* |
| --- | --- | --- |
| Sludges |  |  |
| Aerobic |  |  |
| Control | 0.350 | 0.414 |
| Ag^+^ | 0.029 | 0.064 |
|  |  |  |
| Anaerobic |  |  |
| Control | 0.549 | 0.703 |
| Ag^+^ | 0.015 | 0.042 |
| AgNP | 0.030 | 0.041 |

**References**

[1]F.H. Firsching, S.N. Brune, Solubility products of the trivalent rare-earth phosphates, J. Chem. Eng. Data, 36 (1991) 93-95.

[2]G. Cornelis, C. Doolette, M. Thomas, M.J. McLaughlin, J.K. Kirby, D.G. Beak, D. Chittleborough, Retention and Dissolution of Engineered Silver Nanoparticles in Natural Soils, Soil Sci Soc Am J, 76 (2012) 891-902.
